# Supplementary material for: Investigating the practices and preferences of health scholars in sharing open research data
Source: PLoS One. 2025 Feb 12;20(2):e0313644. doi: 10.1371/journal.pone.0313644 (PMC11819604; doi:10.1371/journal.pone.0313644)
Supplement: Attachment — Submitted filename: Response to Reviewers.docx [file pone.0313644.s001.docx]

Dear

Dr. Bastian Rake

Academic Editor

Plos ONE,

I am writing to resubmit our revised manuscript entitled "Investigating the Practices and Preferences of Health Scholars in Sharing Research Data" for consideration for publication in Plos ONE journal.

We are grateful for the detailed and constructive feedback provided by you and the reviewers. We have carefully addressed all the comments and made the necessary revisions to improve the manuscript.

The major changes made to the manuscript are as follows:

- Reviewer 1 and Reviewer 3 request more clarity regarding the actual meaning “opening” and “sharing” data. I agree that defining these, partially overlapping but different, terms clearly in the beginning of the manuscript will be helpful for readers. In addition, further clarifications regarding whether and how specific practices are really events of data sharing, e.g., sharing within a research team, are required. I think that a clear definition will be helpful to contextualize and interpret these events.

Regarding this request:

To address this request, we have omitted the word "opening" and consistently used "sharing" throughout the manuscript. Sharing to the public can be considered as "opening," so using "sharing" increases the clarity of the article.

- Based on the comments and suggestion made by Reviewer 1 and Reviewer 2, I ask you to expand the concluding section and to discuss your main findings in the context of the existing literature (see also Reviewer 1’ s suggestion for restructuring and moving contents from the Findings). Revising the Conclusion may also help you to emphasize what your study adds to the literature compared to previous studies – an issue that has been raised by Reviewer 3.

Regarding this request:

Based on their comments and suggestions, we expanded the conclusion and discussed our main findings in the context of the prior studies and literature in the last paragraph in the conclusion section.

- Finally, a thorough language editing will be helpful avoid some not well take phrases and to improve the readability of your manuscript.

In addition to the specific changes mentioned above, we have carefully proofread the entire manuscript and made minor adjustments to improve clarity and readability.

Additionally, we added an explanation regarding consent and study exemption in the method section.

The study is funded by the American Indonesian Cultural and Educational Foundation (AICEF) dissertation travel grant and the Florida State University, School of Information, Esther Maglathlin Doctoral Research Scholarship. The funders had no role in study design, data collection and analysis, decision to publish, or preparation of the manuscript.

We believe that these revisions have significantly enhanced the quality of our manuscript and have addressed all the concerns raised by the reviewers. We are confident that the revised manuscript is now suitable for publication in PlosOne Journal.

Thank you for considering our revised manuscript. We look forward to your favorable response. Please let us know if there are any further revisions or additional information required.

Sincerely,

Muhamad Prabu Wibowo

On behalf of Lorraine Mon
