# Supplementary material for: Investigating the practices and preferences of health scholars in sharing open research data
Source: PLoS One. 2025 Feb 12;20(2):e0313644. doi: 10.1371/journal.pone.0313644 (PMC11819604; doi:10.1371/journal.pone.0313644)
Supplement: Attachment — Submitted filename: Response to Reviewers.docx [file pone.0313644.s002.docx]

Dear

Dr. Bastian Rake

Academic Editor

Plos ONE,

I am writing to resubmit our second revised manuscript entitled "Investigating the Practices and Preferences of Health Scholars in Sharing Research Data" for consideration for publication in Plos ONE journal.

We are grateful for the detailed and constructive feedback provided by you and the reviewers. We have carefully addressed all the comments and made the necessary revisions to improve the manuscript.

The major changes made to the manuscript are as follows:

- Reviewer 1 requests re-evaluation of the word “open”. Regarding this request:

We appreciate Reviewer 1’s suggestion regarding the reevaluation of the term “open.” The meanings of sharing and “open” are related. We have re-evaluated the use of the word “open” throughout the manuscript to ensure clarity and precision. However, we have retained the term in certain instances. The distinction between "open" and "share" lies in the degree of accessibility. While sharing can be restricted, open research data refers to making data publicly available while adhering to specific guidelines.

This paper evaluates both data sharing and open research data. Therefore, the use of the term “open” helps explain how these concepts are related. This paper is heavily influenced by Zuiderwijk et al. (2020), in *What drives and inhibits researchers to share and use open research data? A systematic literature review to analyze factors influencing open research data adoption* (*PLoS ONE*, Vol. 15, pp. 1–50), where both terms are used to explain the relationship between data sharing and open data. In response, we have added further explanation in the literature review, expanding on the relationship between data sharing and open research data (lines 107–127).

By retaining the term "open" in certain instances, we hope readers will better understand how these concepts are related.

- Reviewer 3 requests Justification for the Kruskal-Wallis Test. Regarding this request:

We have also addressed Reviewer 3’s request for further justification of the Kruskal-Wallis test in the methods part (line 277 – 281). We have added references to support the appropriateness of the Kruskal-Wallis test for our data, particularly in cases where the data do not meet the assumptions of parametric tests.

We believe that these revisions have significantly enhanced the quality of our manuscript and have addressed all the concerns raised by the reviewers. We are confident that the revised manuscript is now suitable for publication in PlosOne Journal.

Thank you for considering our revised manuscript. We look forward to your favorable response. Please let us know if there are any further revisions or additional information required.

Sincerely,

Muhamad Prabu Wibowo

On behalf of Lorri Mon
